# Supplementary material for: Cumulus cell expansion, nuclear maturation and embryonic development of bovine cumulus-oocyte complexes matured in varying concentrations of follicular fluid
Source: PLoS One. 2025 Feb 7;20(2):e0318376. doi: 10.1371/journal.pone.0318376 (PMC11805436; doi:10.1371/journal.pone.0318376)
Supplement: S1 Table — (DOCX) [file pone.0318376.s001.docx]

| **Supporting Table 1.** List of abbreviations and treatments. | |
| --- | --- |
| Abbreviation | Definition |
| COC | cumulus oocyte complex |
| CC | cumulus cell |
| Mat (h) | oocyte maturation time in hours |
| FF | follicular fluid |
| cOMM | control (conventional) oocyte maturation medium |
| eOMM | experimental oocyte maturation medium |
| FF25 | 25% pooled follicular fluid in eOMM |
| FF50 | 50% pooled follicular fluid in eOMM |
| FF75 | 75% pooled follicular fluid in eOMM |
| FF100 | 100% pooled follicular fluid |
| LFF50 | 50% large follicular fluid in eOMM |
| LFF75 | 75% large follicular fluid in eOMM |
| SFF50 | 50% small follicular fluid in eOMM |
| SFF75 | 75% small follicular fluid in eOMM |
|  |  |
