# Supplementary material for: Cumulus cell expansion, nuclear maturation and embryonic development of bovine cumulus-oocyte complexes matured in varying concentrations of follicular fluid
Source: PLoS One. 2025 Feb 7;20(2):e0318376. doi: 10.1371/journal.pone.0318376 (PMC11805436; doi:10.1371/journal.pone.0318376)
Supplement: Attachment — Submitted filename: Response to Reviewers.docx [file pone.0318376.s002.docx]

Journal requirements:

When submitting your revision, we need you to address these additional requirements.

1.  Please ensure that your manuscript meets PLOS ONE's style requirements, including those for file naming. The PLOS ONE style templates can be found at

[https://journals.plos.org/plosone/s/file?id=wjVg/PLOSOne_formatting_sample_main_body.pdf](https://nam04.safelinks.protection.outlook.com/?url=https%3A%2F%2Ftrack.editorialmanager.com%2FCL0%2Fhttps%3A%252F%252Fjournals.plos.org%252Fplosone%252Fs%252Ffile%253Fid%3DwjVg%252FPLOSOne_formatting_sample_main_body.pdf%2F1%2F010f0194254fe5ff-b4f28899-3e61-4572-bb10-671575e477e5-000000%2FzmVvFw8DFZ_Z2Oalc0src0MzukeblXnMfwhzZmyDZKw%3D191&data=05%7C02%7Crhoadsm%40vt.edu%7Cc90036a7a06546986d9e08dd2ae77a30%7C6095688410ad40fa863d4f32c1e3a37a%7C0%7C0%7C638713896146367791%7CUnknown%7CTWFpbGZsb3d8eyJFbXB0eU1hcGkiOnRydWUsIlYiOiIwLjAuMDAwMCIsIlAiOiJXaW4zMiIsIkFOIjoiTWFpbCIsIldUIjoyfQ%3D%3D%7C0%7C%7C%7C&sdata=IyoQoDFOIMAna5kDbeKcC%2FS41qBugUFK3sBnUpeQ9Kg%3D&reserved=0)  and

[https://journals.plos.org/plosone/s/file?id=ba62/PLOSOne_formatting_sample_title_authors_affiliations.pdf](https://nam04.safelinks.protection.outlook.com/?url=https%3A%2F%2Ftrack.editorialmanager.com%2FCL0%2Fhttps%3A%252F%252Fjournals.plos.org%252Fplosone%252Fs%252Ffile%253Fid%3Dba62%252FPLOSOne_formatting_sample_title_authors_affiliations.pdf%2F1%2F010f0194254fe5ff-b4f28899-3e61-4572-bb10-671575e477e5-000000%2F8miDmndyGbHtSR46bBmLequva0IUf35PpAsrme0iXHo%3D191&data=05%7C02%7Crhoadsm%40vt.edu%7Cc90036a7a06546986d9e08dd2ae77a30%7C6095688410ad40fa863d4f32c1e3a37a%7C0%7C0%7C638713896146382094%7CUnknown%7CTWFpbGZsb3d8eyJFbXB0eU1hcGkiOnRydWUsIlYiOiIwLjAuMDAwMCIsIlAiOiJXaW4zMiIsIkFOIjoiTWFpbCIsIldUIjoyfQ%3D%3D%7C0%7C%7C%7C&sdata=tG8nR9%2BwFv7T%2F0o7eH6F26SzmGVKEgNaeCe2GdeIWVk%3D&reserved=0)

AU: Done

2. Thank you for stating the following financial disclosure:

“Funding for this work was provided, in part, by the Virginia Agricultural Experiment Station and the Hatch Program of the National Institute of Food and Agriculture, U.S. Department of Agriculture.”

Please state what role the funders took in the study.  If the funders had no role, please state: "The funders had no role in study design, data collection and analysis, decision to publish, or preparation of the manuscript."

If this statement is not correct you must amend it as needed.

Please include this amended Role of Funder statement in your cover letter; we will change the online submission form on your behalf.

AU: Done

3. Thank you for stating the following in the Acknowledgments Section of your manuscript:

“The authors would like to express their appreciation to Ali Wood for her dedication to this work and contribution to the manuscript.  Funding for this work was provided, in part, by the Virginia Agricultural Experiment Station and the Hatch Program of the National Institute of Food and Agriculture, U.S. Department of Agriculture.”

We note that you have provided funding information that is currently declared in your Funding Statement. However, funding information should not appear in the Acknowledgments section or other areas of your manuscript. We will only publish funding information present in the Funding Statement section of the online submission form.

Please remove any funding-related text from the manuscript and let us know how you would like to update your Funding Statement. Currently, your Funding Statement reads as follows:

“Funding for this work was provided, in part, by the Virginia Agricultural Experiment Station and the Hatch Program of the National Institute of Food and Agriculture, U.S. Department of Agriculture.”

Please include your amended statements within your cover letter; we will change the online submission form on your behalf.

AU: Done

4. When completing the data availability statement of the submission form, you indicated that you will make your data available on acceptance. We strongly recommend all authors decide on a data sharing plan before acceptance, as the process can be lengthy and hold up publication timelines. Please note that, though access restrictions are acceptable now, your entire data will need to be made freely accessible if your manuscript is accepted for publication. This policy applies to all data except where public deposition would breach compliance with the protocol approved by your research ethics board. If you are unable to adhere to our open data policy, please kindly revise your statement to explain your reasoning and we will seek the editor's input on an exemption. Please be assured that, once you have provided your new statement, the assessment of your exemption will not hold up the peer review process.

5. Please include your full ethics statement in the ‘Methods’ section of your manuscript file. In your statement, please include the full name of the IRB or ethics committee who approved or waived your study, as well as whether or not you obtained informed written or verbal consent. If consent was waived for your study, please include this information in your statement as well.

AU: Done

6. We notice that your supplementary table is included in the manuscript file. Please remove them and upload them with the file type 'Supporting Information'. Please ensure that each Supporting Information file has a legend listed in the manuscript after the references list.

AU: Done

Reviewers' comments:

Reviewer's Responses to Questions

**Comments to the Author**

1. Is the manuscript technically sound, and do the data support the conclusions?

The manuscript must describe a technically sound piece of scientific research with data that supports the conclusions. Experiments must have been conducted rigorously, with appropriate controls, replication, and sample sizes. The conclusions must be drawn appropriately based on the data presented.

Reviewer #1: Yes

Reviewer #2: Yes

AU: Thank you to the reviewers for all of their evaluations!

2. Has the statistical analysis been performed appropriately and rigorously?

Reviewer #1: Yes

Reviewer #2: Yes

AU: Thank you!

3. Have the authors made all data underlying the findings in their manuscript fully available?

The [PLOS Data policy](https://nam04.safelinks.protection.outlook.com/?url=http%3A%2F%2Ftrack.editorialmanager.com%2FCL0%2Fhttp%3A%252F%252Fwww.plosone.org%252Fstatic%252Fpolicies.action%2523sharing%2F1%2F010f0194254fe5ff-b4f28899-3e61-4572-bb10-671575e477e5-000000%2FASjzc5lyMjhXAM1irx-NKPLHrAwNZH9AsvJoeBDaFH4%3D191&data=05%7C02%7Crhoadsm%40vt.edu%7Cc90036a7a06546986d9e08dd2ae77a30%7C6095688410ad40fa863d4f32c1e3a37a%7C0%7C0%7C638713896146392379%7CUnknown%7CTWFpbGZsb3d8eyJFbXB0eU1hcGkiOnRydWUsIlYiOiIwLjAuMDAwMCIsIlAiOiJXaW4zMiIsIkFOIjoiTWFpbCIsIldUIjoyfQ%3D%3D%7C0%7C%7C%7C&sdata=tws%2BEk5qH8qT2jBn7BydOc%2FDGN%2FbxlMaqEw2EuxAoJw%3D&reserved=0) requires authors to make all data underlying the findings described in their manuscript fully available without restriction, with rare exception (please refer to the Data Availability Statement in the manuscript PDF file). The data should be provided as part of the manuscript or its supporting information, or deposited to a public repository. For example, in addition to summary statistics, the data points behind means, medians and variance measures should be available. If there are restrictions on publicly sharing data—e.g. participant privacy or use of data from a third party—those must be specified.

Reviewer #1: No

Reviewer #2: Yes

AU: Data is now available and information is provided on the submission platform and in the cover letter.

4. Is the manuscript presented in an intelligible fashion and written in standard English?

PLOS ONE does not copyedit accepted manuscripts, so the language in submitted articles must be clear, correct, and unambiguous. Any typographical or grammatical errors should be corrected at revision, so please note any specific errors here.

Reviewer #1: Yes

Reviewer #2: Yes

AU: Thank you.

5. Review Comments to the Author

Please use the space provided to explain your answers to the questions above. You may also include additional comments for the author, including concerns about dual publication, research ethics, or publication ethics. (Please upload your review as an attachment if it exceeds 20,000 characters)

Reviewer #1: The manuscript is well written. The language is generally clear. However, I think some minor adjustments or corrections need to be made to improve the article.

AU: We wish to thank the reviewer for taking the time to evaluate our submission!

On line 36 to 38, that is the abstract section, you wrote "In the final two experiments".... Are they actually two separate experiments? I think you need to rewrite that sentence to improve clarity.

AU: The reviewer is correct – thank you for pointing out our mistake. The verbiage indicating two final experiments was left over from an old draft where the experiments were described differently. We have revised accordingly.

I think you need to improve the clarity of line 100 to 105. In line 104, you wrote "and adapted to use as base".... That sentence is not very clear.

AU: Thank you for pointing this out. We have revised to improve the clarity.

Line 224 to 225, That sentence is not necessary in the result section. You have already mentioned the aim in the introduction.

AU: Thank you for this suggestion. The sentence has been removed.

The first two paragraphs of the discussion section are absolutely not necessary. I think you should discuss your findings and briefly explain how they relate or differ from similar studies. You only gave an overview of the literature, and that is something to be done in the introduction section. The same applies to the first few sentences of the third paragraph. The correct discussion only started in line 307. I think you need to remove the first two and half paragraphs in the discussion and improve on the discussion.

AU: Thank you for this suggestion. We agree with the reviewer’s assessment and have revised this portion significantly. The quality of the discussion is greatly improved as a result of this suggestion.

Reviewer #2: No comment for the authors. The topic is novel and the languish is sound. The article can be accepted in the current form.
All questions above were answered . No additional comments for the author, including concerns about dual publication, research ethics, or publication ethics. (Please upload your review as an attachment if it exceeds

AU: Thank you very much for taking the time to evaluate our submission!

6. PLOS authors have the option to publish the peer review history of their article ([what does this mean?](https://nam04.safelinks.protection.outlook.com/?url=https%3A%2F%2Ftrack.editorialmanager.com%2FCL0%2Fhttps%3A%252F%252Fjournals.plos.org%252Fplosone%252Fs%252Feditorial-and-peer-review-process%2523loc-peer-review-history%2F1%2F010f0194254fe5ff-b4f28899-3e61-4572-bb10-671575e477e5-000000%2Fv1pJ7kLjvJDeksYhre0JDsDy4k6UpAz-Tcyvpbzdp2Y%3D191&data=05%7C02%7Crhoadsm%40vt.edu%7Cc90036a7a06546986d9e08dd2ae77a30%7C6095688410ad40fa863d4f32c1e3a37a%7C0%7C0%7C638713896146402333%7CUnknown%7CTWFpbGZsb3d8eyJFbXB0eU1hcGkiOnRydWUsIlYiOiIwLjAuMDAwMCIsIlAiOiJXaW4zMiIsIkFOIjoiTWFpbCIsIldUIjoyfQ%3D%3D%7C0%7C%7C%7C&sdata=hYxJGsW9tc%2FPzOq9%2FTrHUlqs%2BVK5li%2FXRKH4EbbENeo%3D&reserved=0)). If published, this will include your full peer review and any attached files.


If you choose “no”, your identity will remain anonymous but your review may still be made public.


**Do you want your identity to be public for this peer review?** For information about this choice, including consent withdrawal, please see our [Privacy Policy](https://nam04.safelinks.protection.outlook.com/?url=https%3A%2F%2Ftrack.editorialmanager.com%2FCL0%2Fhttps%3A%252F%252Fwww.plos.org%252Fprivacy-policy%2F1%2F010f0194254fe5ff-b4f28899-3e61-4572-bb10-671575e477e5-000000%2F5lPrBucl3HD8QfK5XoA0su5Qsg_0CBVwXO6hC6qLpdY%3D191&data=05%7C02%7Crhoadsm%40vt.edu%7Cc90036a7a06546986d9e08dd2ae77a30%7C6095688410ad40fa863d4f32c1e3a37a%7C0%7C0%7C638713896146411985%7CUnknown%7CTWFpbGZsb3d8eyJFbXB0eU1hcGkiOnRydWUsIlYiOiIwLjAuMDAwMCIsIlAiOiJXaW4zMiIsIkFOIjoiTWFpbCIsIldUIjoyfQ%3D%3D%7C0%7C%7C%7C&sdata=EfhfWyR%2F4QesHEgVISpCG2o5nUSb3rBrT2n3DTl6zYo%3D&reserved=0).

Reviewer #1: No

Reviewer #2: **Yes:** Amal Mahmoud Aboelmaaty
